# 

**Published:** 1901-08-17

**Authors:** 


### NOTICE TO CORRESPONDENTS.

All MSS., Letters, Books for Review, and other matters intended for the Editor should be addressed The Editor, "The Hospital," The Hospital Building, 28 & 29 Southampton Street, Strand, W.C.
The Editor cannot undertake to return rejected MS., even when accompanied by stamped directed envelope.

**Notice to Advertisers and Subscribers.**

All Advertisements, Orders for Copies of The Hospital, and Business Communications should be addressed to **THE MANAGER (Not to the Editor),** The Hospital, 28 & 29 Southampton Street, Strand, London, W.C.

The Cost of Subscription, post free, is as follows:—Per week, 2½d.; per quarter, 2s. 8d.; per half-year, 5s. 3d.; per annum, 10s. 6d. Foreign:—Per annum, 15s. 2d., payable, in all cases, in advance.

**NOTICE.—Vol. XXIX. of "The Hospital" (October 1900, to March, 1901), in handsome cloth binding is now on sale at the Office of this Journal, price, 7s. 6d. Cases for binding the Numbers contained in this Volume 1s. 6d. Index to Vol. XXIX., 2½d., post free.**

**The Monthly Part for August is now ready. Price 6d., by post 8d.**

### BOOKS RECEIVED.

Baillière, Tindall and Cox.
"Typhoid, the Destroyer of Armies, and its Abolition." By Leigh Cawney, M.D. (Lond.).

James Nisbet and Co
"A Woman's Memories of the War." By Violet Brooke-Hunt.

Smith, Elder, and Co.
"A Retrospect of Surgery during the past Century." By John Poland, F.R.C.S.

Longmans, Green and Co.
"Invalid Recipes." By E. E. Mann.

Borough Printing Works.
"The Government and Old Age Pensions."

Cassell and Co.
"Tumours, Innocent and Malignant." By J. Bland-Sutton.

Bailliere, Tindall and Cox.

"Diseases of the Stomach and their Surgical Treatment." By A. W. Mayo Robson, F.R.C.S., and B. G. A. Moynihan, M.S Lond., F.R.C.S.

performed; but we learn from a note that anæsthetics were administered 115 times. The medical and surgical tables merely name the diseases and give the number of patients who suffered from each disease. There were 21 cases of enteric fever, but we are not told how many of these died; and so on all through the list of diseases treated during the year.

### Scraps and Gleanings.

The summer fête held on behalf on the Gravesend Hospital produced the sum of £509. This success was greatly due to the efforts of the Ladies' Committee.

His Majesty the King has consented to become the patron of the Birmingham and Midland Ear and Throat Hospital, of which Lord Leigh has been president for the last 40 years.

It is proposed that the Victoria Memorial at Kidderminster shall take the form of various important alterations and additions to the infirmary, estimated to cost about £3,000.

The Hop Picking Mission Committee appeal for help to send out in the coming season some twenty-five workers amongst the hoppers in mid-Kent —all volunteers, including eight nurses.

The expenditure last year at the Gravesend Hospital was £3,402, and the revenue £2,884. There were 567 in-patients during the year compared with only 480 the year before. There was also an increase of 360 out-patients during the year.

Hospital Saturday in Gravesend this year will be on September 28th. In view of this, collection sheets have already been printed and will be provided to those workshops applying for them with the object of securing contributions of a penny a week.

During the month of June six patients were discharged from the home of the Society for the Prevention and Cure of Consumption in the county of Durham. All the patients are doing well: the new south block is now in occupation, and the new wing is progressing satisfactorily.

The new consumptive hospital for Perth and Perthshire, which has been built and equipped by Sir Robert and Lady Pullar, contains accommodation for twenty patients, one wing being for men and one for women. It is hoped to get a sum of £10,000 to enable eight of the beds to be endowed.

At a garden party held recently in honour of the members of the Ladies' Executive Collecting Committee of the York County Hospital, it was announced that the result of these collections was that a sum of £1,827 had been received, of which £682 odd was from the city, £422 odd from the county, and £182 odd in donations.

At the close of last year the deficit in the revenue of the North Riding Infirmary, Middlesborough, amounted to £1,087, and at the close of the present year it is expected it will be double this sum. It is clear, therefore, that either the work must be curtailed or the income must be permanently increased by an amount equal to upwards of £1,000 per year. It is stated that the casualty cases coming from elsewhere than the works are what mainly cause the annual deficits, and the workmen are appealed to on this account to increase their subscriptions, as, although not coming from the works, these are in effect workmen's cases.

The report of Mr. Macpherson upon the proposed cottage hospital at Monifieth, N.B., to be erected by the trustees of the late Dr. Young, was recently submitted to the first division of the Court of Session. This report states that certain modifications of the plans have been suggested by the architect, which will increase the cost of the proposed hospital from £1,850 to £2,080. The trustees had consented to this, and there was the further question to decide as to whether, in view of the population of Monifieth, it would not be wise to increase the accommodation proposed immediately, which enlargement would cost £200 or £300. The report is to come up for consideration in October.

### The Student of Medicine.

### APPOINTMENTS.

**Richard J. A. Berry**, M.D., F.R.S.E., appointed Examiner in Anatomy to the University of Aberdeen. **William Butler**, M.B.Glasg., C.M., D.P.H.R.C.P.S.Lond., appointed Medical Superintendent, Isolation Hospital, Willesden, and Assistant Medical Officer of Health to the Willesden Urban District Council. **R. H. Dix**, M.B., B.S., appointed Junior House Physician to the Royal Infirmary, Newcastle-on-Tyne. **Duncan Forbes**, M.D., B.Sc., appointed Assistant Medical Officer of Health for Leith. **J. Harley Gough**, F.R.C.S., appointed Certifying Factory Surgeon for the Torquay District. **J. W. Heslop**, M.B., B.S., appointed House Surgeon to the Royal Infirmary, Newcastle-on-Tyne. **Clara Hind**, L.R.C.P. and S. Edin. and Glasg., appointed Resident Medical Officer to the Rudgwick Sanatorium, Sussex. **William M. P. Keogh**, M.B., B.Ch., B.A.O.R.U.I., appointed Assistant Medical Officer County Asylum, Newport, Isle of Wight. **R. H. Meikle**, M.B., M.S.Glasg., appointed Medical Officer of Health for the Darlington Rural District. **H. Carden Pearson**, M.B., F.R.C.S.Edin., appointed Surgeon to the Darlington Hospital. **A. E. Porter**, M.D., D.P.H.Camb., appointed Assistant Medical Officer of Health to the City of Leeds. **Marion Ethel Unwin**, L.R.C.P. and S.Edin. and Glasg., appointed Junior Resident Medical Officer to the Clapham Maternity Hospital.

ON SOME ASPECTS OF MEMORY.
By John Aikman, M.D.
Reprinted from "The Hospital." Crown 8vo., paper cover, price 6d.
THE SCIENTIFIC PRESS, LTD., 28 & 29 Southampton Street, Strand, London, W.C.

